# Supplementary material for: Acquired miR-142 deficit in leukemic stem cells suffices to drive chronic myeloid leukemia into blast crisis
Source: Nat Commun. 2023 Sep 1;14:5325. doi: 10.1038/s41467-023-41167-z (PMC10474062; doi:10.1038/s41467-023-41167-z)
Supplement: Supplementary file 1 — Supplementary Information [file 41467_2023_41167_MOESM1_ESM.pdf]

Acquired miR-142 Deficit in Leukemic Stem Cells Suffices to Drive Chronic Myeloid Leukemia into Blast Crisis

Inventory of Supplementary Information

Supplementary figure 1

Supplementary figure 2

Supplementary figure 3

Supplementary figure 4

Supplementary figure 5

Supplementary figure 6

Supplementary figure 7

Supplementary figure 8

Supplementary figure 9

Supplementary figure 10

Supplementary Table 1

Supplementary Table 2

Supplementary Table 3

Supplementary Table 4

Supplementary Fig. 1

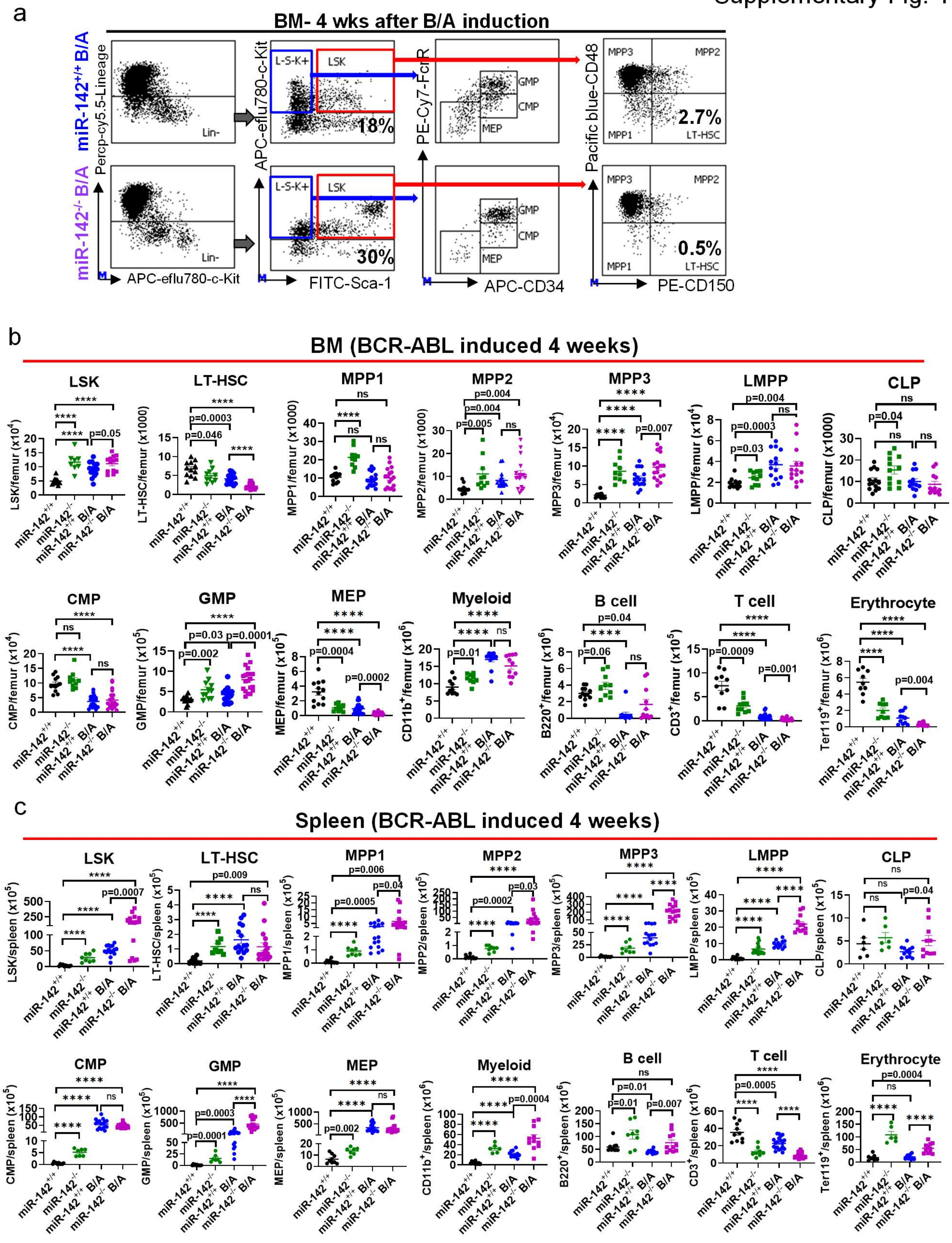

Supplementary Fig. 1: BM and spleen hematopoietic cell subpopulations in *miR-142*$^{-/-}$, *miR-142*$^{+/+}$, *miR-142*$^{-/-}$*BCR-ABL*, and *miR-142*$^{+/+}$*BCR-ABL* mice. a Representative plots of BM subpopulations, i.e., LSK (Lin$^-$Sca-1$^+$c-Kit$^+$), GMP (Lin$^-$Sca-1$^-$c-Kit$^+$ CD34$^+$FcγRII/III^{hi}), CMP (Lin$^-$Sca-1$^-$c-Kit$^+$ CD34$^+$FcγRII/III^{low}), MEP (Lin$^-$Sca-1$^-$c-Kit$^+$ CD34$^{low/-}$FcγRII/III$^{low/-}$), LT-HSC (LSK Flt3$^-$CD150$^+$CD48$^-$), MPP1 (LSK Flt3$^-$CD150$^-$CD48$^-$), MPP2 (LSK Flt3$^-$CD150$^+$CD48$^+$), MPP3 (LSK Flt3$^-$CD150$^-$CD48$^+$) in gender- and age-matched *miR-142*$^{-/-}$ and *miR-142*$^{+/+}$ non-leukemic mice and *miR-142*$^{-/-}$*BCR-ABL* and *miR-142*$^{+/+}$*BCR-ABL* leukemic mice (measured at 4 weeks after BCR-ABL induction by tetracycline withdrawal). **b** Numbers of BM LSK, LT-HSC, MPP1, MPP2, MPP3, LMPP (LSK Flt3$^+$CD150$^-$), CLP (Flt3$^+$IL-7Rα$^+$Lin$^-$Sca-1^{low}c-Kit^{low}), CMP, GMP, MEP, myeloid (CD11b$^+$Gr-1$^+$), B (B220$^+$), T (CD3$^+$) and erythroid (Ter119$^+$) cells from one femur of gender- and age-matched *miR-142*$^{-/-}$ and *miR-142*$^{+/+}$ non-leukemic mice (n=10 mice per group) and *miR-142*$^{-/-}$*BCR-ABL* and *miR-142*$^{+/+}$*BCR-ABL* leukemic mice (measured at 4 weeks after BCR-ABL induction by tetracycline withdrawal, n=12 mice per group). **c** Numbers of spleen LSK, LT-HSC, MPP1, MPP2, MPP3, LMPP, CLP, CMP, GMP, MEP, myeloid, B, T and erythroid cells in the gender- and age-matched *miR-142*$^{-/-}$ and *miR-142*$^{+/+}$ non-leukemic mice (n=10 mice per group) and *miR-142*$^{-/-}$*BCR-ABL* and *miR-142*$^{+/+}$*BCR-ABL* leukemic mice (measured at 4 weeks after BCR-ABL induction by tetracycline withdrawal, n=12 mice per group). For **b-c**, source data are provided as a Source Data file. For **a,** results from one of the three independent experiments are shown (n=3). Abbreviations: *miR-142*$^{+/+}$*B/A*: *miR-142*$^{+/+}$*BCR-ABL*; *miR-142*$^{-/-}$*B/A*: *miR-142*$^{-/-}$*BCR-ABL;* BM: bone marrow; LT-HSC: long-term hematopoietic stem cells; MPP: multipotent progenitor; LMPP: lymphoid-primed MPP; CLP: common lymphoid progenitor; CMP: common myeloid progenitor; GMP: granulocyte/monocyte progenitor; MEP: megakaryocyte/erythroid progenitor. Comparison between groups was performed by two-tailed, unpaired t-test. Results shown represent mean ± SEM (standard error of the mean). Significance values: ****, $p<0.0001$; ns: not significant.

Supplementary Fig. 2

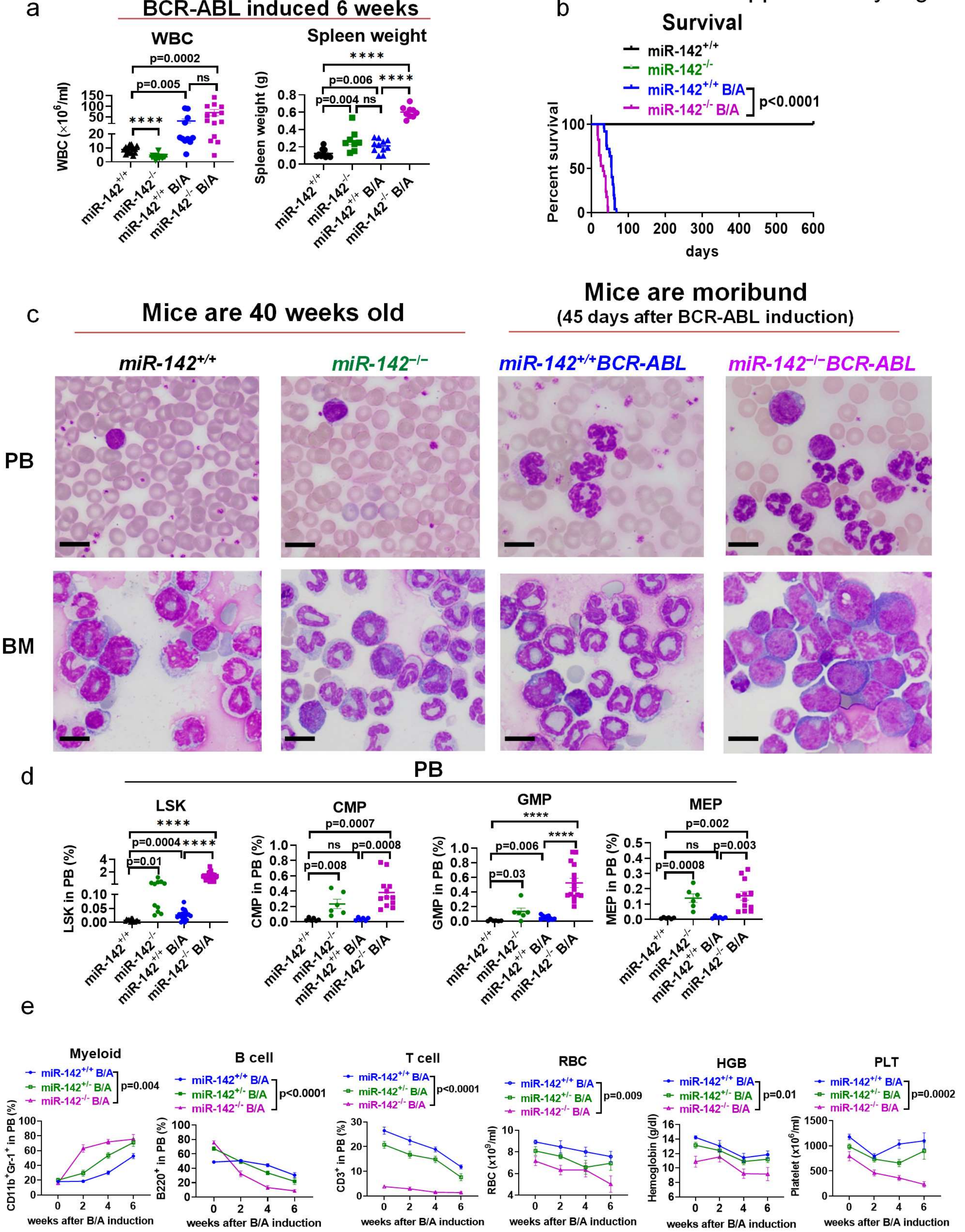

Supplementary Fig. 2: Blood hematopoietic cell subpopulations in *miR-142*$^{-/-}$, *miR-142*$^{+/+}$, *miR-142*$^{-/-}$*BCR-ABL*, and *miR-142*$^{+/+}$*BCR-ABL* mice. **a** White blood cell (WBC) counts (n=16 mice per group) and spleen weight (n=9 mice per group) of gender- and age-matched *miR-142*$^{-/-}$ and *miR-142*$^{+/+}$ non-leukemic mice and *miR-142*$^{-/-}$*BCR-ABL* and *miR-142*$^{+/+}$*BCR-ABL* leukemic mice, measured at 6 weeks after BCR-ABL induction by tetracycline withdrawal. **b** Survival of gender- and age-matched *miR-142*$^{-/-}$ and *miR-142*$^{+/+}$ non-leukemic mice (n=10 per group) and *miR-142*$^{-/-}$*BCR-ABL* and *miR-142*$^{+/+}$*BCR-ABL* leukemic mice (upon tetracycline withdrawal to induce BCR-ABL expression, n=17 per group). **c** Blood and BM smears from 40-week-old *miR-142*$^{-/-}$ and *miR-142*$^{+/+}$ non-leukemic mice and from moribund *miR-142*$^{-/-}$*BCR-ABL* and *miR-142*$^{+/+}$*BCR-ABL* leukemic mice at 45 days after BCR-ABL induction by tetracycline withdrawal, assessed by microscopy. Scale bar: 10μM. The experiment was repeated three times independently with similar results. **d** Percentage of PB LSK (Lin^{-}Sca-1^{+}c-Kit^{+}), CMP (Lin^{-}Sca-1^{-}c-Kit^{+} CD34^{+}FcγRII/III^{low}), GMP (Lin^{-}Sca-1^{-}c-Kit^{+} CD34^{+}FcγRII/III^{hi}), and MEP (Lin^{-}Sca-1^{-}c-Kit^{+} CD34$^{low/-}$FcγRII/III$^{low/-}$) in gender- and age-matched *miR-142*$^{-/-}$ and *miR-142*$^{+/+}$ non-leukemic mice (n=6 per group) and *miR-142*$^{-/-}$*BCR-ABL* (n=12) and *miR-142*$^{+/+}$*BCR-ABL* (n=6) leukemic mice (measured at 4 weeks after BCR-ABL induction by tetracycline withdrawal), analyzed by flow cytometry. **e** PB myeloid (CD11b^{+}Gr-1^{+}), B (B220^{+}) and T (CD3^{+}) cell percentages by flow cytometry analysis, and red blood cell (RBC) counts, hemoglobin (HGB) and platelet (PLT) counts by HESKA-element-HT5, in gender-, age-, and BCR-ABL-level-matched *miR-142*$^{-/-}$*BCR-ABL* (n=10), *miR-142*$^{+/-}$*BCR-ABL* (n=11) and *miR-142*$^{+/+}$*BCR-ABL* (n=15) mice measured before (0 weeks) and 2, 4 and 6 weeks after BCR-ABL induction by tetracycline withdrawal. For **a-b** and **d-e**, source data are provided as a Source Data file. For **c**, results from one of the three independent experiments are shown (n=3). Abbreviations: *miR-142*$^{+/+}$*B/A*: *miR-142*$^{+/+}$*BCR-ABL*; *miR-142*$^{-/-}$*B/A*: *miR-142*$^{-/-}$*BCR-ABL;* PB: peripheral blood; CMP: common myeloid progenitor*;* GMP: granulocyte/monocyte progenitor; MEP: megakaryocyte/erythroid progenitor. Comparison

between groups was performed by two-tailed, unpaired t-test. Results shown represent mean ± SEM (standard error of the mean). Significance values: ****, $p<0.0001$, ns: not significant.

Supplementary Fig. 3

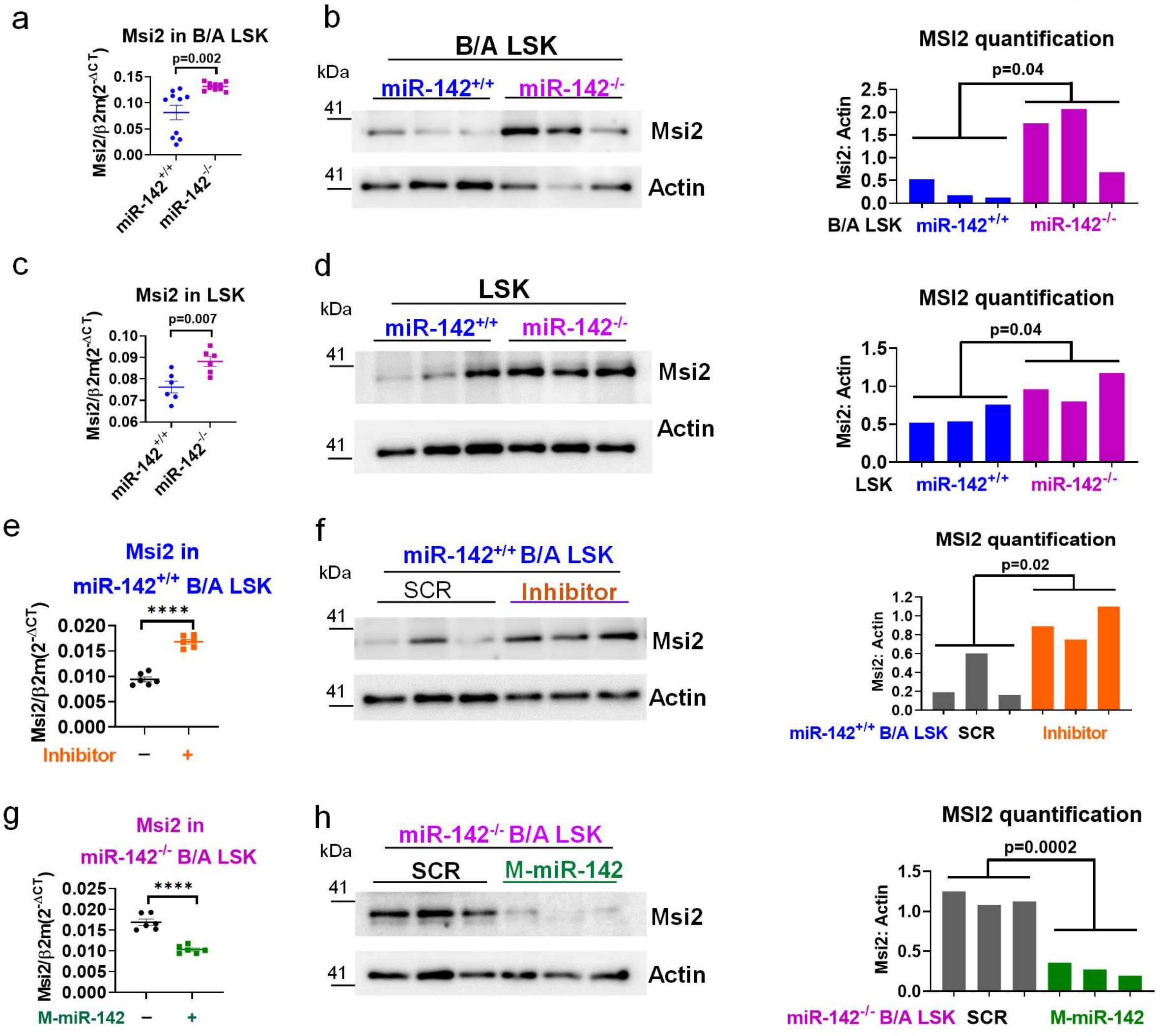

i

4343-4350 of **human** MSI2 3' UTR:	5'	CCGUCCUGGGCACAGACACUACA
4442-4449 of **mouse** MSI2 3' UTR:	5'	CAGUCCUGGGCACAGACACUACA
		| | | | | | | |
hsa- and mmu-miR-142a-3p.1	3'	AGGUAUUUCAUCCUUUGUGAUGU
4075-4082 of **human** MSI2 3' UTR:	5'	AUUGUUUACAAAAGAAACACUAA
4145-4152 of **mouse** MSI2 3' UTR:	5'	AUUGUUUACAAAAGAAACACUAA
		| | | | | | | |
hsa- and mmu-miR-142a-3p.2	3'	AGGUAUUUCAUCCUUUGUGAUG

Supplementary Fig. 3: MSI2 is a target of miR-142-3p. a-b Msi2 mRNA (**a**, n=10 biologically independent samples) and protein (**b**; left, western blot results; right, MSI2 protein quantification; n=3 biologically independent samples) levels in LSKs from miR-142$^{-/-}$BCR-ABL (BC CML) mice compared with those from miR-142$^{+/+}$BCR-ABL (CP CML) mice. Source data are provided as a Source Data file. **c-d** Msi2 mRNA (**c**, n=6 biologically independent samples) and protein (**d**; left, western blot results; right, MSI2 protein quantification; n=3 biologically independent samples) levels in LSKs from miR-142$^{-/-}$ mice compared with those from miR-142$^{+/+}$ mice. Source data are provided as a Source Data file. **e-f** Msi2 mRNA (**e**, n=6 biologically independent samples) and protein (**f**; left, western blot results; right, MSI2 protein quantification; n=3 biologically independent samples) levels in LSKs from miR-142$^{+/+}$BCR-ABL mice treated *in vitro* with miR-142 inhibitor (CpG-anti-miR-142 ODN) that downregulated miR-142 levels. Source data are provided as a Source Data file. **g-h** Msi2 mRNA (**g**, n=6 biologically independent samples) and protein (**h**; left, western blot results; right, MSI2 protein quantification; n=3 biologically independent samples) levels in LSKs from miR-142$^{-/-}$BCR-ABL mice treated *in vitro* with miR-142 mimics (CpG-M-miR-142) that upregulated miR-142 levels. Source data are provided as a Source Data file. **i** A conserved 8mer miR-142-3p-binding site on the 3' UTR of human and mouse MSI2, highlighted in yellow, was shown by TargetScan (8.0). For **a-h**, source data are provided as a Source Data file. For **b, d, f, h**, results from one of the three independent experiments are shown (n=3). Abbreviations: B/A: BCR-ABL; LSK: Lin-Sca-1+c-Kit+ cells. Comparison between groups was performed by two-tailed, unpaired t-test. Results shown represent mean ± SEM (standard error of the mean). Significance values: ****, $p<0.0001$.

Supplementary Fig. 4

a

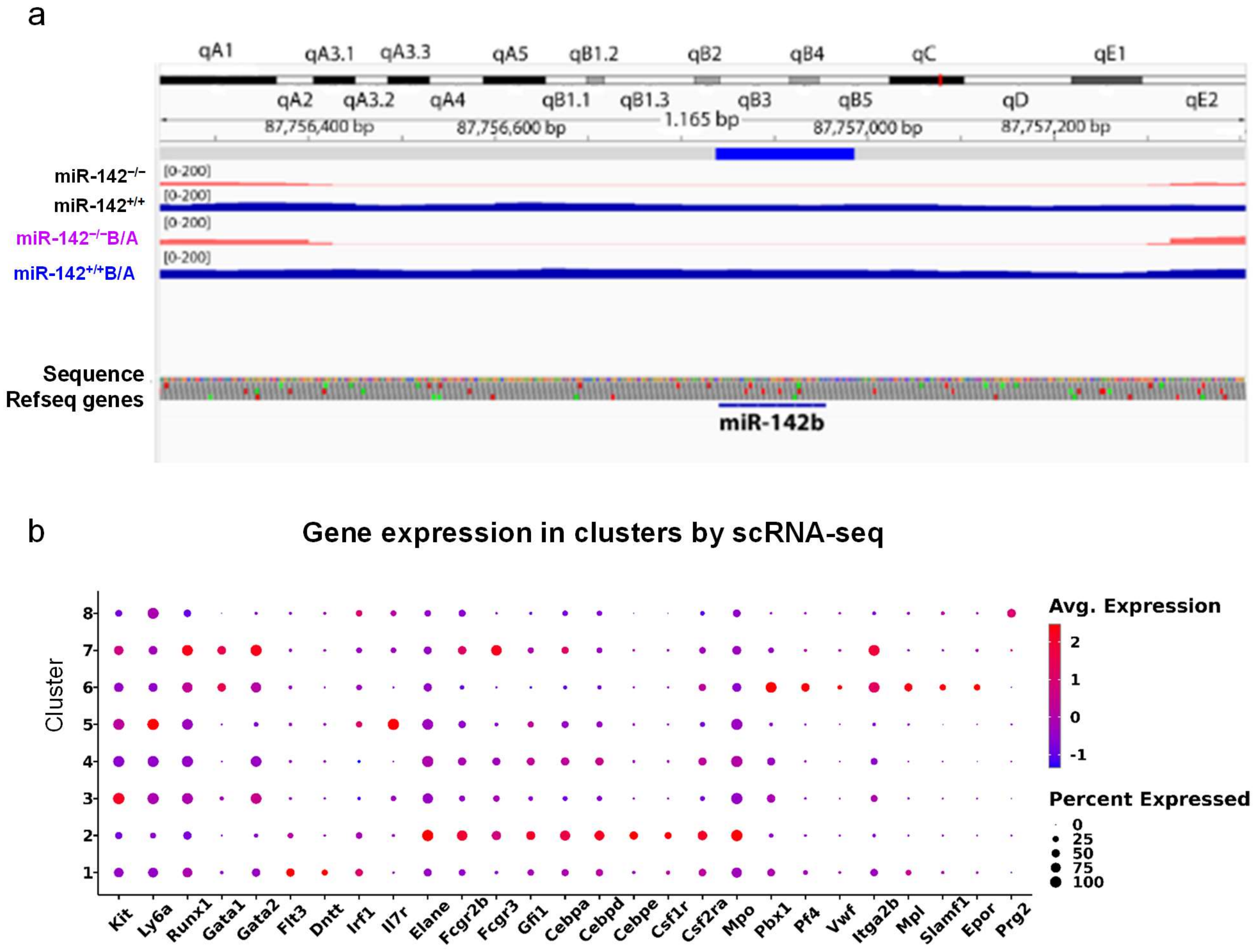

c

Cluster identification of scRNA-seq and gene markers

Cell Type	Cluster	Gene markers
Lymphoid-primed HSC	1	Flt3, Dntt, Irf1
Myeloid/neutrophil-primed HSC	2	Elane, Fcgr2b (CD32), Fcgr3 (CD16), Gfi1, Cebpa, Cepbd, Cebpe, Csf1r, Csf2ra, Mpo
Non-primed-1 HSC	3	Gata2, Kit^{high}, Ly6a^{high}, Runx1
Myeloid-primed HSC	4	Elane, Fcgr2b (CD32), Fcgr3 (CD16), Gfi1, Cebpa, Cebpd, Csf2ra
Myeloid/lymphoid-primed HSC	5	Elane, Il7r
Megakaryocyte-Erythrocyte-primed HSC	6	Pbx1, Pf4, Gata1, Gata2, Vwf, Itga2b (CD41), Mpl, Slamf1 (CD150), Epor
Non-primed-2 HSC	7	Gata1, Gata2, Runx1, Itga2b(CD41)
Eosinophil-primed HSC	8	Prg2

Supplementary Fig. 4: DNA-sequencing and cluster identification of scRNA-seq of BM LSKs from *miR-142*$^{-/-}$*BCR-ABL* vs *miR-142*$^{+/+}$*BCR-ABL* mice. a Integrative genomics viewer (IGV) screenshot showing deletion of miR-142 loci in *miR-142*$^{-/-}$ and *miR-142*$^{-/-}$*BCR-ABL* LSKs. Topmost bar is the copy number variant (CNV) summary track. LSK samples from *miR-142*$^{-/-}$ and *miR-142*$^{-/-}$*BCR-ABL mice* are shown in red bars and LSKs from *miR-142*$^{+/+}$ and *miR-142*$^{+/+}$*BCR-ABL* mice are shown in blue bars. The deleted CNV encompasses Mir142b. **b** Expression levels of selected genes in clusters identified by scRNA-seq. **c** Gene markers selected for each cluster and cluster identification of scRNA-seq. Abbreviations: *B/A*: *BCR-ABL*; scRNA-seq: single cell RNA sequencing.

Supplementary Fig. 5

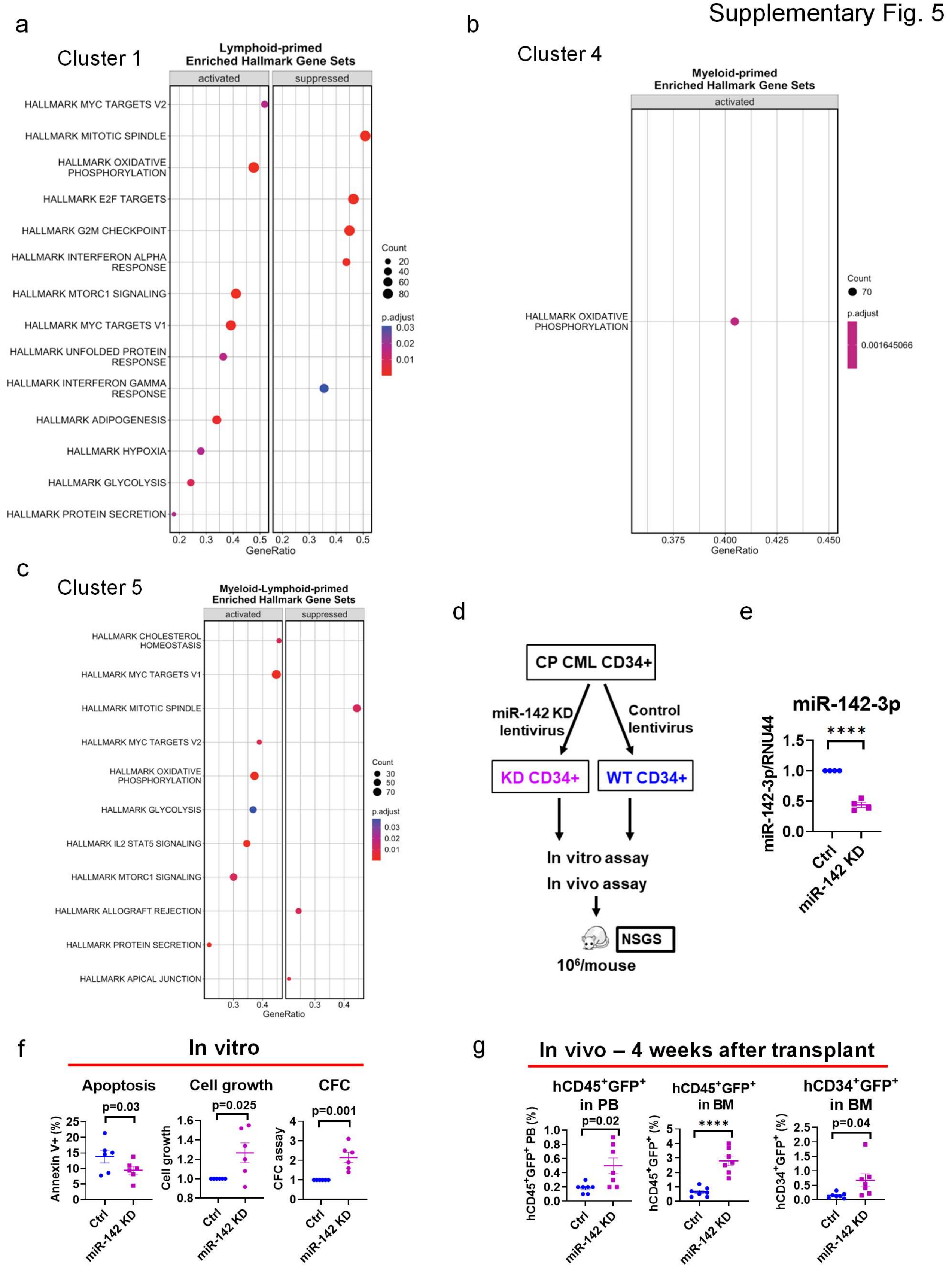

Supplementary Fig. 5: GSEA of *miR-142$^{-/-}$BCR-ABL* vs *miR-142$^{+/+}$BCR-ABL* LSK clusters and *in vitro* and *in vivo* characterization of miR-142 KD primary CML CD34^{+} cells. a-c GSEA was performed on differentially expressed genes in clusters 1 (**a**), 4 (**b**) and 5 (**c**) from scRNA-seq of BM LSKs from *miR-142$^{-/-}$BCR-ABL and miR-142$^{+/+}$BCR-ABL* mice (BCR-ABL induced for 2 weeks). **d** Experimental design. **e** miR-142 expression in human CP CML CD34^{+} cells transduced with GFP-expressing miR-142 KD or control lentiviruses (n=4 independent experiments). **f** Apoptosis, cell growth and colony forming cells (CFC) (n=6 independent experiments for all) in GFP^{+} miR-142 KD CD34^{+} cells or control CD34^{+} cells after *in vitro* culture for 72 hours. **g** GFP^{+} miR-142 KD CD34^{+} CML cells or control cells were transplanted into immunodeficient NSG mice. Blood and BM engraftment rates (human CD45^{+}GFP^{+} cells) and BM LSC-enriched cell engraftment rates (human CD45^{+}CD34^{+}GFP^{+} cells) were shown (n=7 mice per group). For **d-g**, source data are provided as a Source Data file. For **e-f**, results from one of the three independent experiments are shown (n=3). Abbreviations: GSEA: gene set enrichment analysis; CP: chronic phase; BM: bone marrow; GFP: green fluorescent protein; KD: knockdown; LSC: leukemia stem cells. Comparison between groups was performed by two-tailed, unpaired t-test. Results shown represent mean ± SEM (standard error of the mean). Significance values: ****, $p<0.0001$.

Supplementary Fig. 6

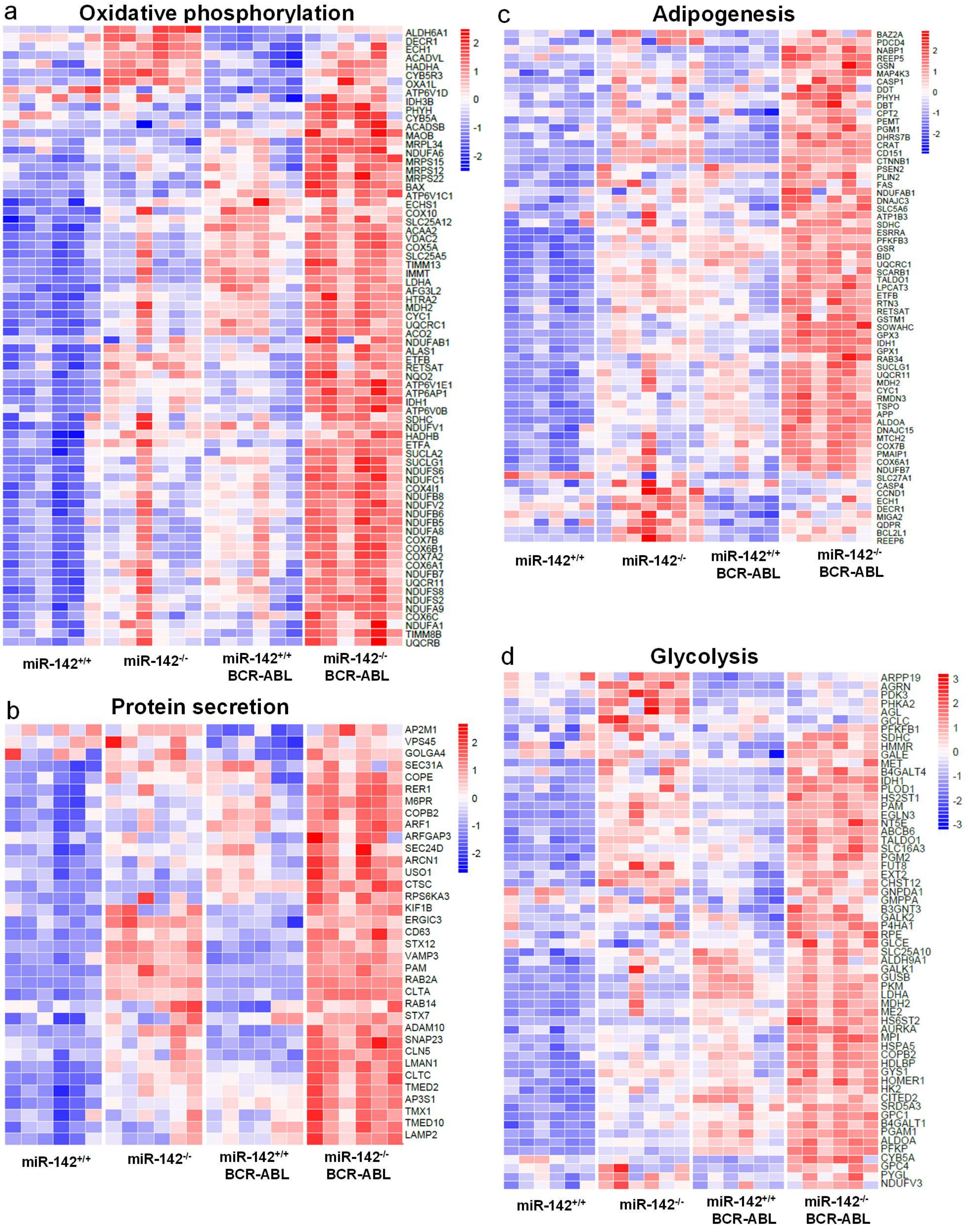

Supplementary Fig. 6: Heatmap showing differentially expressed genes from the top four upregulated pathways in BC CML (*miR-142$^{-/-}$BCR-ABL*) LSKs vs CP CML (*miR-142$^{+/+}$BCR-ABL*) LSKs. a-d Heatmap of differentially expressed genes from the top four upregulated pathways in *miR-142$^{-/-}$BCR-ABL* LSKs vs *miR-142$^{+/+}$BCR-ABL* LSKs, i.e., oxidative phosphorylation (OxPhos; **a**), protein secretion (**b**), adipogenesis (**c**), and glycolysis (**d**) in BM LSKs from *miR-142$^{+/+}$* (wt), *miR-142$^{-/-}$* (miR-142 KO), *miR-142$^{+/+}$BCR-ABL* (CP CML) and *miR-142$^{-/-}$BCR-ABL* (BC CML) mice (n=6 mice per group). Abbreviations: CP: chronic phase; BC: blast crisis; BM: bone marrow; KO: knockout.

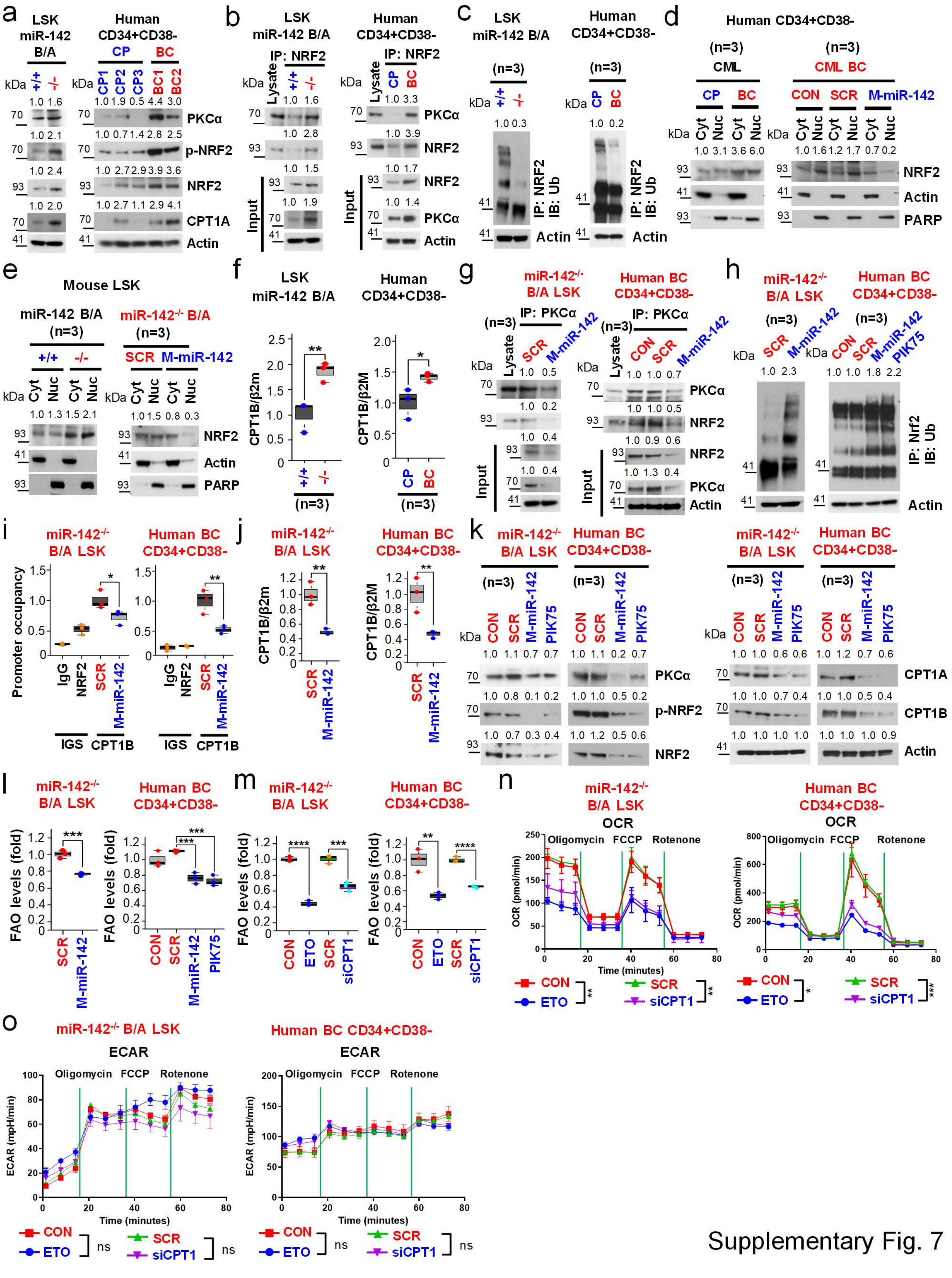

Supplementary Fig. 7

Supplementary Fig. 7: Regulation of PKCα/NRF2/CPT1A-B axis by miR-142 in LSC-enriched cells. a Effects of miR-142 expression on levels of PKCα, p-NRF2, and NRF2 protein. Lysates from *miR-142*$^{+/+}$ vs *miR-142*$^{-/-}$*BCR-ABL* LSKs (**left**) or human CP vs BC CD34^{+}CD38^{-} CML cells (**right**) were immunoblotted with the indicated antibodies. Densitometry quantifications are shown by number (fold) on the top of blots. **b-c** Effects of miR-142 expression on NRF2/PKCα binding and NRF2 ubiquitination. Indicated lysates were subjected to immunoprecipitation with anti-NRF2 antibody and immunoblotted with anti-PKCα (**b**) or anti-ubiquitin (**c**) antibody. Three samples of each group were pooled for the assay (n=3). **d-e** Effects of miR-142 expression on cellular distribution of NRF2. Cellular fractionation was performed using CD34^{+}CD38^{-} cells from human CP vs BC CML patients (**d, left**) and LSKs from *miR-142*$^{+/+}$*BCR-ABL* vs *miR-142*$^{-/-}$*BCR-ABL* mice (**e, left**), or human BC CML CD34^{+}CD38^{-} cells (**d, right**) and mouse *miR-142*$^{-/-}$*BCR-ABL* LSKs (**e, right**) treated with SCR control or CpG-M-miR-142 (2 μM) for 24 hours. Indicated lysate was immunoblotted with anti-NRF2, anti-actin and anti-PARP antibodies. Three samples of each group were pooled for the assay (n=3). For **a-e**, results from one of the two independent experiments are shown. **f** Effects of miR-142 expression on levels of CPT1B mRNA (**left**, $p = 0.0076$; **right**, $p = 0.024$). Related to Figure **5c**. mRNA was extracted from cells described in **b** and **c** and CPT1B mRNA was detected by qPCR. Three samples of each group were pooled for the assay (n=3). **g-l** Effects of correcting miR-142 deficit on PKCα/NRF2/CPT1B signaling and FAO levels. Mouse *miR-142*$^{-/-}$*BCR-ABL* LSK (**left**) and human BC CML CD34^{+}CD38^{-} (**right**) cells were treated with SCR, CpG-M-miR-142 (2 μM), or PKCα inhibitor-PIK75 (1 μM) for 24 hours. **g** Levels of PKCα/NRF2 interaction by immunoprecipitation. **h** Levels of NRF2 ubiquitination. **i-j** Levels of NRF2/CPT1B promoter binding by ChIP assay (**left**, $p = 0.033$; **right**, $p = 0.0085$) and CPT1B mRNA by q-PCR (**left**, $p = 0.0027$; **right**, $p = 0.0084$). Box plot with median value and first/third quartiles and whiskers together with minimum and maximum values are shown. **k** Protein expression levels of PKCα, p-NRF2, NRF2, and CPT1A/B by immunoblotting. **l** Significant reduction in the levels of FAO were measured by the oxidation rate of 3H-palmitic acid (**left**, *p* =

0.0006; **right**, SCR vs M-miR-142, *p = 0.0007* and SCR vs PIK75, *p = 0.0003*). For **g-l**, three samples of each group were pooled for the assay (n=3). **m-o** Effects of CPT1 inhibitor etomoxir (ETO) and CPT1A/B knockdown on FAO/OXPHOS. Mouse *miR-142*$^{-/-}$*BCR-ABL* LSK (**left**) and human BC CML CD34^{+}CD38^{-} (**right**) cells were treated with DMSO vs ETO (5μM) or siSCR vs siCPT1A/B (each, 20nM) for 24 hours (n=3 biologically independent samples). **m** Levels of FAO were measured by the oxidation rate of 3H-palmitic acid (**left**, CON vs ETO, *p = 0.00001* and SCR vs siCPT1, *p = 0.0007*; **right**, CON vs ETO, *p = 0.0033* and SCR vs siCPT1, *p = 0.00004*). **n-o** Levels of OCR and ECAR were measured by Seahorse XF Cell Mito Stress Test assay (**n**: **left**, CON vs ETO, *p = 0.0077* and SCR vs siCPT1, *p = 0.0023*; **right**, CON vs ETO, *p = 0.0142* and SCR vs siCPT1, *p = 0.0001*; **o:** not significant). For **a-o**, source data are provided as a Source Data file and results from one of the three independent experiments are shown (n=3). Abbreviations: LSCs: leukemia stem cells; LSK; Lin^{-}Sca-1^{+}c-Kit^{+}; miR-142$^{+/+}$B/A: *miR-142*$^{+/+}$*BCR-ABL*; miR-142$^{-/-}$B/A: *miR-142*$^{-/-}$*BCR-ABL*; CP: chronic phase; BC: blast crisis; ChIP; chromatin immunoprecipitation; FAO: fatty acid oxidation; OXPHOS: oxidative phosphorylation; M-miR-142: CpG-M-miR-142; Cyt: cytoplasm; Nuc: nucleus; ETO: etomoxir; DMSO: dimethyl sulfoxide; siCPT1: siCPT1A/B; OCR: oxygen consumption rate; ECAR: extracellular acidification rate; SCR: scramble; CON: control. Comparison between groups was performed by one-tailed, unpaired t-test. Results shown represent mean ± SD (standard deviation). Significance values: *, $p < 0.05$; **, $p < 0.01$; ***, $p < 0.001$; ****, $p < 0.0001$; ns, not significant.

Supplementary Fig. 8

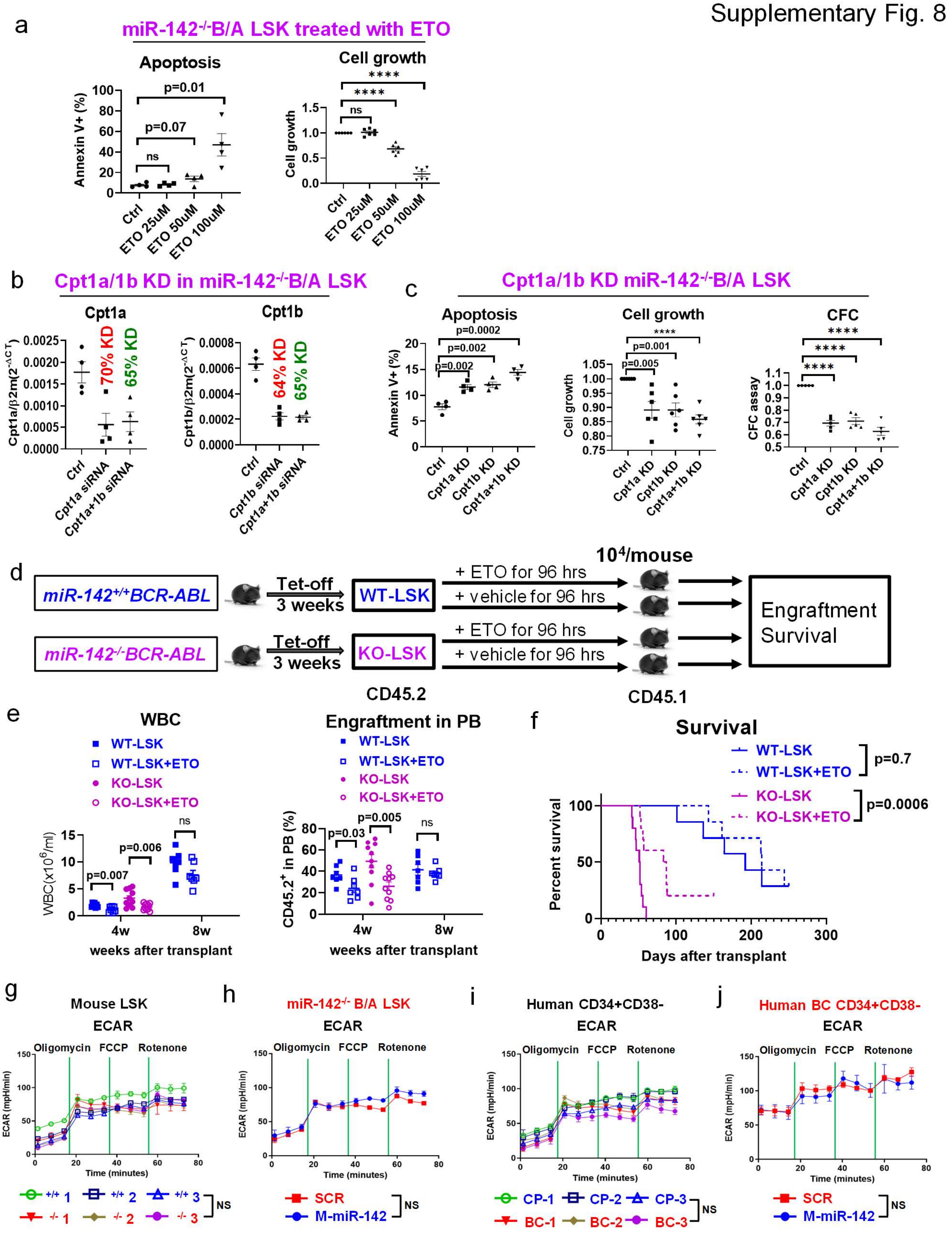

Supplementary Fig. 8: Inhibition of FAO reduced self-renewal activity of LSC-enriched cells. a-c *miR-142*$^{-/-}$*BCR-ABL* LSKs (CPT1^{high}) were treated with the CPT1 inhibitor ETO for 72 hours (**a**) or CTP1A/1B siRNAs to knock down CTP1A/1B (**b-c**). Cpt1a and Cpt1b levels by Q-RT-PCR (n=4 independent experiments), apoptosis by flow cytometry (n=4 independent experiments), cell growth (n=6 independent experiments), and/or colony-forming cells (CFC; n=5 independent experiments) were shown. **d** LSKs selected from *miR-142*$^{+/+}$*BCR-ABL* or *miR-142*$^{-/-}$*BCR-ABL* mice 3 weeks after BCR-ABL induction were treated ex vivo with ETO (50μM) or vehicle for 96 hours and then transplanted into congenic wt B6 mice via tail vein injection. **e-f** WBC counts, circulating engraftment rates at 4 weeks (**e**), and survival (**f**) of the recipients of ETO-treated *miR-142*$^{-/-}$*BCR-ABL* LSKs compared to recipients of vehicle-treated *miR-142*$^{-/-}$*BCR-ABL* LSKs (n=10 mice per group) were shown. **g-j** Levels of glycolysis (as indicated by ECAR levels) in LSKs isolated from *miR-142*$^{+/+}$*BCR-ABL* vs *miR-142*$^{-/-}$*BCR-ABL* mice and human CD34^{+}CD38^{-} cells from CP vs BC CML patients (**g** and **i**), or in murine *miR-142*$^{-/-}$*BCR-ABL* LSK and human BC CML CD34^{+}CD38^{-} cells treated with SCR control or CpG-M-miR-142 (2 μM) (**h** and **j**). The levels of ECAR were measured by Seahorse XF Cell Mito Stress Test assay. Relative to Fig. **5d** and **5e**. For **a-c** and **e-j**, source data are provided as a Source Data file. For **g-j**, results from one of the three independent experiments are shown (n=3). Abbreviations: LSK; Lin^{-}Sca-1^{+}c-Kit^{+}; ETO: etomoxir; CP: chronic phase; BC: blast crisis; WBC: white blood cells; ECAR: extracellular acidification rate; SCR: scramble. Comparison between groups was performed by two-tailed, unpaired t-test. Results shown represent mean ± SEM. Significance values: ****, p<0.0001; ns: not significant.

Supplementary Fig. 9

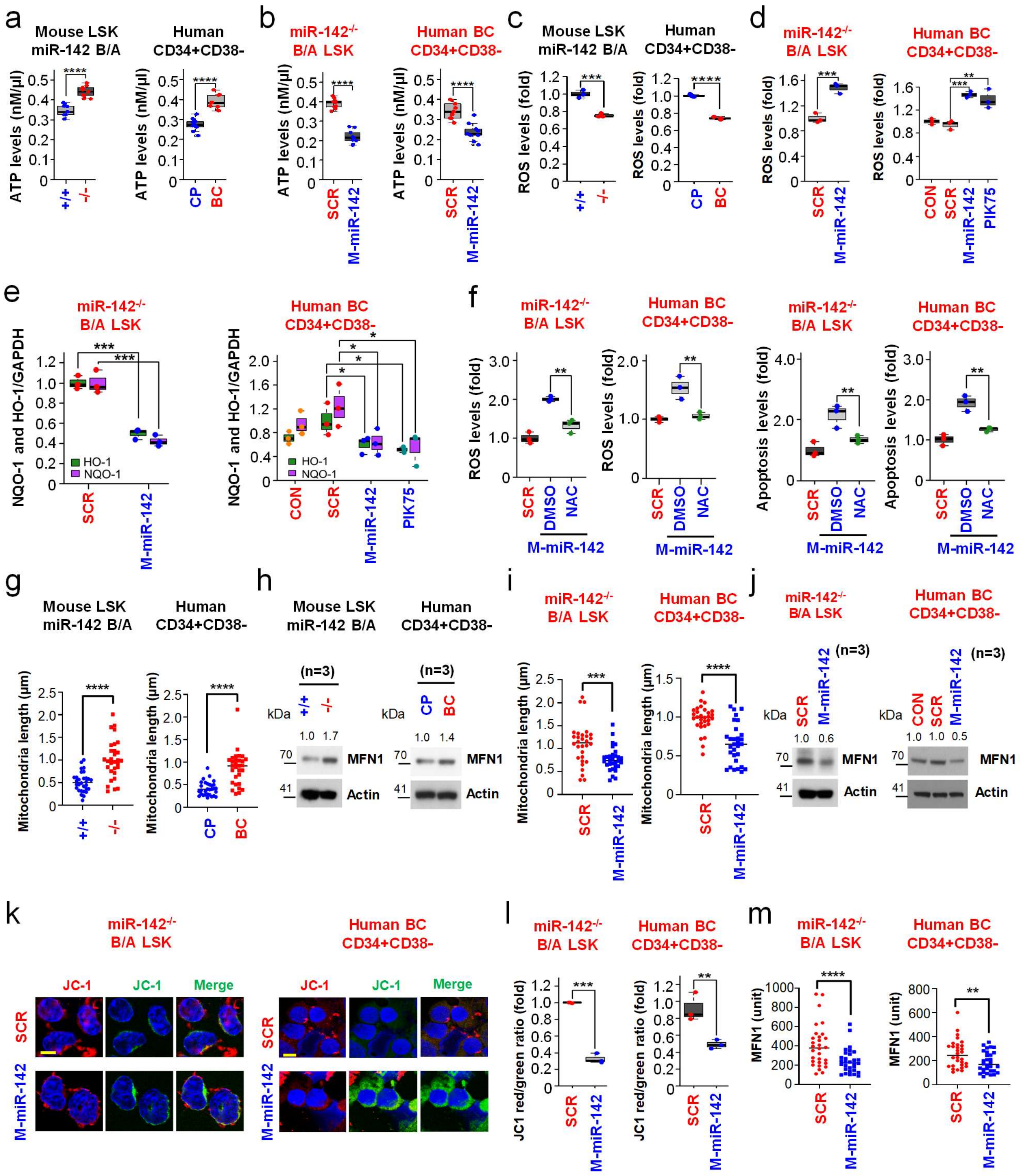

Supplementary Fig. 9: Regulation of ROS levels and mitochondria fusion by miR-142 in LSC-enriched cells. a-b Effects of miR-142 expression on ATP levels. **a** LSKs and $CD34^+CD38^-$ cells were respectively isolated from *miR-142*$^{+/+}$*BCR-ABL* vs *miR-142*$^{-/-}$*BCR-ABL* mice or from human CP vs BC CML patients. **b** Mouse *miR-142*$^{-/-}$*BCR-ABL* LSKs (**left**) or human $CD34^+CD38^-$ BC CML cells (**right**) were treated with SCR control or CpG-M-miR-142 (2 μM) for 24 hours. **a-b** Levels of ATP were measured by ATP assay (**a: left**, *p=2.00E-06*; **right**, *p=1.78E-06*; **b: left**, *p=1.37E-06*; **right**, *p=4.36E-06*). **c-e** Effects of miR-142 expression on NRF2-regulated HO-1/NQO-1 and ROS levels. **c** LSKs and $CD34^+CD38^-$ cells were respectively isolated from *miR-142*$^{+/+}$*BCR-ABL* vs *miR-142*$^{-/-}$*BCR-ABL* mice or from human CP vs BC CML patients. **d,e** Mouse *miR-142*$^{-/-}$*BCR-ABL* LSKs (**left**) or human $CD34^+CD38^-$ BC CML cells (**right**) were treated with SCR control, CpG-M-miR-142 (2 μM), or PIK75 (1μM) for 24 hours. **c,d** ROS levels were measured by flow cytometry with mitoSox staining (**c: left**, *p=0.0005*; **right**, *p=1.78E-05*); (**d: left**, *p=0.0009*; **right**, SCR vs M-miR-142, *p=0.0003* and SCR vs PIK75, *p=0.0071*). **e** HO-1/NQO-1 mRNA was measured by q-PCR (**Left:** HO-1, *p=0.0007* and NQO-1, *p=0.0007*; **Right:** HO-1, SCR vs M-miR-142, *p=0.0451* and SCR vs PIK75, *p=0.0195*; NQO-1, SCR vs M-miR-142, *p=0.0339* and SCR vs PIK75, *p=0.0286*). **f** Effects of *N*-acetyl-l-cysteine (NAC) on M-miR-142 increased ROS and apoptosis. Mouse *miR-142*$^{-/-}$*BCR-ABL* LSKs (**left**) or human $CD34^+CD38^-$ BC CML cells (**right**) were treated with SCR control or CpG-M-miR-142 (2 μM) in the presence of DMSO or NAC (1mM) for 24 hours. Levels of ROS (**left**, mouse LSK, *p=0.0014* and human $CD34^+CD38^-$ cells, *p=0.0074*) and apoptosis (**right**, mouse LSK, *p=0.0015* and human $CD34^+CD38^-$ cells, *p=0.0024*) were measured by flow cytometry with mitoSox and annexin V staining. **a-f** Box plot with median value and first/third quartiles and whiskers together with minimum and maximum values are shown. **g-j** Effects of miR-142 expression on mitochondria fusion and levels of MFN1 protein. **g,h** LSKs (**left**) and $CD34^+CD38^-$ cells (**right**) were respectively isolated from *miR-142*$^{+/+}$*BCR-ABL* vs *miR-142*$^{-/-}$*BCR-ABL* mice or from human CP vs BC CML patients. **i,j** Mouse *miR-142*$^{-/-}$*BCR-ABL* LSKs (**left**) or human $CD34^+CD38^-$ BC CML cells (**right**)

were treated with SCR control or CpG-M-miR-142 (2 μM) for 24 hours. **g,i** Quantification of mitochondria length **(g: left**, *p=6.29E-07*; **right**, *p=1.67E-07*; **i: left**, *p=0.0006*; **right**, *p=2.26E-07*). Related to Fig. **5f-i**. **h,j** Cell lysates were immunoblotted with anti-MFN1 and anti-actin antibodies. Three samples of each group were pooled for the assay (n=3). Densitometry quantifications are shown by number (fold) on the top of blots. **k-l** Effects of miR-142 expression on mitochondrial membrane potential. Murine *miR-142*$^{-/-}$*BCR-ABL* LSK (**left**) and human BC CML CD34^{+}CD38^{-} cells (**right**) were treated with SCR or CpG-M-miR-142 (2 μM) for 24 hours. Mitochondrial membrane potential was measured by staining of the treated cells with JC-1 dye. JC-1 dye exhibits potential-dependent accumulation in mitochondria, indicated by a green fluorescence emission at (~529 nm) for the monomeric form of the probe, which shifts to red (~590 nm) with a concentration-dependent formation of red fluorescent J-aggregates. Mitochondrial depolarization is indicated by a decrease in the red/green fluorescence intensity ratio. **k** Represent images of JC-1 staining. Scale bar, 10 μm. **l** Quantification of JC-1 (red/green) fluorescence intensity (**left**, *p=1.92E-05*; **right**, *p=0.0072*). Box plot with median value and first/third quartiles and whiskers together with minimum and maximum values are shown. **m** Effects of miR-142 expression on MFN1 expression. Murine *miR-142*$^{-/-}$*BCR-ABL* LSK (**left**) and human BC CML CD34^{+}CD38^{-} cells (**right**) were treated with SCR or CpG-M-miR-142 (2 μM) for 24 hours. MFN1 protein was visualized by images of immunolabeling-electron microscope (Fig. **5j-k**) and quantification of MFN1 expression was shown by counting from 30 mitochondria in random fields (**left**, *p=7.34E-04*; **right**, *p=0.0046*). For **a-j** and **l-m**, source data are provided as a Source Data file. For **a-m**, results from one of the three independent experiments are shown (n=3). Abbreviations: LSCs: leukemia stem cells; ATP: adenosine 5′-triphosphate; LSK: Lin^{-}Sca-1^{+}c-Kit^{+}; miR-142$^{+/+}$B/A: *miR-142*$^{+/+}$*BCR-ABL*; miR-142$^{-/-}$B/A: *miR-142*$^{-/-}$*BCR-ABL*; CP: chronic phase, BC: blast crisis; M-miR-142: CpG-M-miR-142; ROS: reactive oxygen species; NAC: *N*-acetyl-l-cysteine; SCR: scramble; DMSO: dimethyl sulfoxide. Comparison between groups was

performed by one-tailed, unpaired t-test. Results shown represent mean ± SD. Significance values: *, $p < 0.05$; **, $p < 0.01$; ***, $p < 0.001$; ****, $p < 0.0001$; ns, not significant.

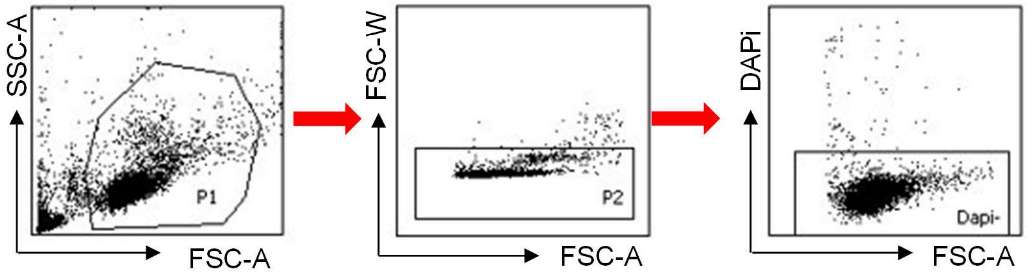

Supplementary Fig. 10: Gating strategy for flow cytometry analysis. All samples were FSC-A and SSC-A gated, followed by FSC-A/FSC-W gating to select singlet cells. DAPI negative cells were then gated for subsequent relevant gating, shown in the main figures or supplementary figures.

Supplementary table 1. Comparison of hematopoietic cell subpopulations in PB, BM and spleen in miR-142 KO BCR-ABL vs miR-142 WT BCR-ABL, and in miR-142 KO vs WT *mice.*

		miR-142$^{-/-}$ B/A vs miR-142$^{+/+}$ B/A			miR-142$^{-/-}$ vs miR-142$^{+/+}$		
**Cells**	**Immunopheotypes**	**PB**	**BM**	**Spleen**	**PB**	**BM**	**Spleen**
**LSK**	Lineage (Lin)$^-$Sca-1$^+$c-Kit$^+$	↑	↑	↑	↑	↑	↑
**LT-HSC**	Flt3$^-$CD150$^+$CD48$^-$ LSK	n/a	↓	=	n/a	↓	↑
**MPP1**	Flt3$^-$CD150$^-$CD48$^-$ LSK	n/a	=	↑	n/a	↑	↑
**MPP2**	Flt3$^-$CD150$^+$CD48$^+$ LSK	n/a	=	↑	n/a	↑	↑
**MPP3**	Flt3$^-$CD150$^-$CD48$^+$ LSK	↑	↑	↑	↑	↑	↑
**LMPP**	Flt3$^+$CD150$^-$ LSK	↑	=	↑	n/a	↑	↑
**CLP**	IL-7Rα$^+$Lin$^-$Sca-1^{low}c-Kit^{low}	n/a	=	↑	n/a	↑	=
**CMP**	Lin$^-$Sca-1$^-$c-Kit$^+$ CD34$^+$FcγRII/III^{low}	↑	=	=	↑	=	↑
**GMP**	Lin$^-$Sca-1$^-$c-Kit$^+$ CD34$^+$FcγRII/III^{hi}	↑	↑	↑	↑	↑	↑
**MEP**	Lin$^-$Sca-1$^-$c-Kit$^+$ CD34$^{low/-}$FcγRII/III$^{low/-}$	↑	↓	=	↑	↓	↑
**Myeloid**	CD11b+Gr-1+	↑	=	↑	↑	↑	↑
**B**	B220+	↓	=	↑	=	=	↑
**T**	CD3+	↓	↓	↓	↓	↓	↓
**Erythrocyte**	Ter119+	↓	↓	↑	↓	↓	↑

LT-HSC: long term hematopoietic stem cell; MPP: multipotent progenitors; LMPP: lymphoid-primed MPP; CLP: common lymphoid progenitor; CMP: common myeloid progenitor; GMP: granulocyte-macrophage progenitor; MEP: megakaryocyte-erythrocyte progenitor; B/A: BCR-ABL; Arrows represent relative cell type levels in miR-142$^{-/-}$ B/A vs miR-142$^{+/+}$ B/A mice or in miR-142$^{-/-}$ vs miR-142$^{+/+}$ mice.

Supplementary Table 2: Summary of compounds detected in untargeted metabolomics of miR-142−/−BCR-ABL (BC CML) and miR-142+/+BCR-ABL (CP CML) study cohort.

	HILICpos	HILICneg	RPpos	RPneg	Combined
Compounds	1577	1116	3696	5010	11399
Annotated	546	327	826	425	2124
PoolQC(Area)	≤25%CV	≤25%CV	≤25%CV	≤25%CV	-
Mass error (ppm)	±5ppm	±5ppm	±5ppm	±5ppm	-
RT tolerance (min)	≤0.2 min	≤0.2 min	≤0.2 min	≤0.2 min	-

Supplementary Table 3: Metabolic ratios of miR-142−/−BCR-ABL (KO) and miR-142+/+BCR-ABL (WT) cells. Ratios were compared using a two-sided Student's t-test.

Comparison	Mean KO Ratio	Mean WT Ratio	KO/WT	p value
NAD+/NADH	1.8095722	0.745690113	2.426708048	0.01842
GSSG/GSH	0.261395837	0.89881819	0.290821704	0.07313

Supplementary Table 4. Reagents or resources used in this study.

Antibodies for flow cytometry analysis

**Anti-human antibodies**				
**Name**	**color**	**manufacturer**	**clone**	**cat**
CD34	PE-Cy7	eBioscience	4H11	25-0349-42
CD34	FITC	Invitrogen	4H11	11-0349-42
CD34	APC	Becton Dickenson	4H11	17-0349-42
CD38	PE	Becton Dickenson	HIT2	12-0389-42
CD38	APC	Becton Dickenson	HB7	340439
CD38	APC-eflu780	eBioscience	HIT2	47-0389-42
CD45	APC	Becton Dickenson	HI30	555485
CD45	FITC	Becton Dickenson	HI30	304038
CD45	APC-eflu780	eBioscience	30-F11	47-0451-82
CD33	PE	Becton Dickenson	WM53	555450
**Anti-mouse antibodies**				
**Name**	**color**	**manufacturer**	**clone**	**cat**
CD3e	biotinylated	eBioscience	145-2c11	13-0031-85
CD4	biotinylated	eBioscience	GK1.5	13-0041-85
CD8a	biotinylated	eBioscience	53-6.7	13-0081-85
B220	biotinylated	eBioscience	RA3-6B2	13-0452-85
CD19	biotinylated	eBioscience	eBio1D3 (1D3)	13-0193-85
IgM	biotinylated	eBioscience	II/41	13-5790-85
Gr-1	biotinylated	eBioscience	RB6-8C5	13-5931-85
CD11b	biotinylated	eBioscience	M1/70	13-0112-85
NK1.1	biotinylated	eBioscience	PK136	13-5941-85
Ter119	biotinylated	eBioscience	TER-119	13-5921-85
Flt3	biotinylated	eBioscience	A2F10	13-1351-85
IL-7Rα	biotinylated	eBioscience	A7R34	13-1271-85
Sca-1	PE–Cy7	eBioscience	D7	25-5981-82
Sca-1	PE	eBioscience	D7	12-5981-83
Sca-1	FITC	eBioscience	D7	11-5981-85
CD117 (c-Kit)	APC-efluor780	eBioscience	ACK2	47-1172-82
CD117 (c-Kit)	APC-efluor780	eBioscience	2B8	47-1171-82
CD16/CD32	PE–Cy7	eBioscience	93	25-0161-82
CD16/CD32	PE-Cy7	Becton Dickenson	2.4G2	560829
CD34	eFluor 660	eBioscience	RAM34	50-0341-82
CD34	Alexa647	biolegend	MEC14.7	119314
CD150	PE	eBioscience	mshad150	12-1502-82
CD150	PE	Biolegend	TC15-12F12.2	115904

CD150	PerCP-Cy5.5	Biolegend	TC15-12F12.2	115922
CD48	APC	eBioscience	HM48-1	17-0481-82
CD48	Pacific blue	Biolegend	HM48-1	103418
FLT3	PE	Invitrogen	A2F10	12-1351-82
FLT3	BV421	Biolegend	A2F10	135315
IL-7Rα	BV421	Biolegend	A7R34	135027
IL-7Rα	FITC	eBioscience	A7R34	11-1271-82
CD45	APC-efluor780	eBioscience	30-F11	47-0451-80
CD45	FITC	eBioscience	30-F11	11-0451-85
CD45	PE	Invitrogen	30-F11	12-0451-82
Gr-1	FITC	eBioscience	RB6-8C5	11-5931-85
CD11b	PE	eBioscience	M1/70	12-0112-83
CD3	APC-efluor780	Invitrogen	I7A2	47-0032-82
B220	FITC	Invitrogen	RA3-6B2	11-0452-82
Ter119	APC-efluor780	eBioscience	TER-119	47-5921-82
Ter119	eFluor 450	eBioscience	TER-119	48-5921-82
CD45.1	PE-Cy7	eBioscience	A20	25-0453-82
CD45.1	PE	eBioscience	A20	12-0453-83
CD45.2	FITC	eBioscience	104	11-0454-85
CD45.2	APC	eBioscience	104	11-0454-82
**Other antibodies**				
Annexin V	PE	Becton Dickenson		559763
Ki-67	Alexa Fluor 647	Becton Dickenson	B56	551126
streptavidin	PE	eBioscience		12-4317-87
streptavidin	FITC	eBioscience		11-4317-87
streptavidin	APC	Invitrogen		17-4317-82
streptavidin	Efluro 450	ebiosience		48-4317-82

Taqman assays for Q-RT-PCR

Gene name	Assay ID	Company
RNU44	1094	ThermoFisher
snoRNA234	1234	ThermoFisher
miR-142-3p	464	ThermoFisher
miR-142-5p	2248	ThermoFisher
Cpt1a	Mm01231183_m1	ThermoFisher
Cpt1b	Mm00487191_g1	ThermoFisher
Msi2	Mm01304232_m1	ThermoFisher
β2m	Mm00437762_m1	ThermoFisher
CPT1B	Hs03046298_s1	ThermoFisher

β2M	Hs00187842_m1	ThermoFisher

Primer sequences for Q-PCR

Gene name	primer	sequence
Human NQO-1	F	GCCGCAGACCTTGTGATATT
	R	TTTCAGAATGGCAGGGACTC
Mouse NQO-1	F	TTCTGTGGCTTCCAGGTCTT
	R	AGGCTGCTTGGAGCAAAATA
Human HO-1	F	ATGGCCTCCCTGTACCACATC
	R	TGTTGCGCTCAATCTCCTCCT
Mouse HO-1	F	CCTTCCCGAACATCGACAGCC
	R	GCAGCTCCTCAAACAGCTCAA
Human GAPDH	F	CCCCTTCATTGACCTCAACTACAT
	R	CGCTCCTGGAAGATGGTGA
Mouse GAPDH	F	GAGCCAAAAGGGTCATCATC
	R	TAAGCAGTTGGTGGTGCAGG
Human CPT1B		VMPS-1352, RealtimePrimers.com
Mouse Cpt1b		VHPS-2185, RealtimePrimers.com

Primer sequences for CHIP assay

Gene name	primer	sequence
Human CPT1B	Pro-F	CCCCGTGCTGTGTATGTAAC
	Pro-R	GTGTGTACTGCTCGGTTTGG
Human IGS	Pro-F	TAAGACCCTGAACCGAAGGA
	Pro-R	TCCCCCTTCAAAACACAAAC
Mouse Cpt1b	Pro-F	AACCTTGAGCCCTGGAATTAG
	Pro-R	ACGTGAGCATGGTTGCAT
Mouse Igs	Pro-F	CAGTCCACGCAGTAACTTGT
	Pro-R	CTTTCTTGGTGGCTCCAGTC

Antibodies for immunoprecipitation and immunoblotting analyses

Anti- antibody	manufacturer	cat
Nrf2	Abcam	ab137550
PARP	Cell signaling	# 9542
Actin	Santa Cruz	sc-47778
Ubiquitin	Millipore	07-375
MFN1	Cell signaling	# 14739

Tom20	Santa Cruz	sc-17764
CPT1A	Abcam	ab128568
CPT1B	Abcam	ab134988
PKCα	Santa Cruz	sc-8393
p-NRF2	Cell Signaling	# 12721
MSI2	ThermoFisher	10770-1-AP

Other reagents

**Reagents**	**manufacturer**	**cat**
NAC	Selleckchem	#S5804
SiCPT1A (human)	Horizon	ID# M-009749-02-0005
SiCPT1B (human)	Horizon	ID# M-010266-01-0005
siCPT1A (mouse)	Horizon	ID# E-042456-00-0005
siCPT1B (mouse)	Horizon	ID# E-043173-00-0005
