# Supplementary material for: Acquired miR-142 deficit in leukemic stem cells suffices to drive chronic myeloid leukemia into blast crisis
Source: Nat Commun. 2023 Sep 1;14:5325. doi: 10.1038/s41467-023-41167-z (PMC10474062; doi:10.1038/s41467-023-41167-z)
Supplement: Supplementary file 4 — Description of Additional Supplementary Files [file 41467_2023_41167_MOESM4_ESM.pdf]

Description of Additional Supplementary Files

Supplementary Data 1
Description: Total compounds and their relative abundances in miR-142−/−BCR-ABL vs miR-142+/+BCR-ABL Lin-c-Kit+ cells

Supplementary Data 2
Description: Metabolic changes in Lin-c-Kit+ cells from miR-142−/−BCR-ABL (BC CML) mice compared with Lin-c-Kit+ cells from miR-142+/+ BCR-ABL (CP CML) mice
